# An Unavoidable Mind-Set Reversal: Consciousness in Vision Science

**DOI:** 10.3390/brainsci14070735

**Published:** 2024-07-22

**Authors:** Liliana Albertazzi

**Affiliations:** University of Trento, 38122 Trento, Italy; lili.albertazzi@gmail.com

**Keywords:** consciousness, experimental phenomenology, first person account, neuroscience, self-referentiality, visual perception

## Abstract

In recent decades, the debate on consciousness has been conditioned by the idea of bottom-up emergence, which has influenced scientific research and raised a few obstacles to any attempt to bridge the explanatory gap. The analysis and explanation of vision conducted according to the accredited methodologies of scientific research in terms of physical stimuli, objectivity, methods, and explanation has encountered the resistance of subjective experience. Moreover, original Gestalt research into vision has generally been merged with cognitive neuroscience. Experimental phenomenology, building on the legacy of Gestalt psychology, has obtained new results in the fields of amodal contours and color stratifications, light perception, figurality, space, so-called perceptual illusions, and subjective space and time. Notwithstanding the outcomes and the impulse given to neuroscientific analyses, the research carried out around these phenomena has never directly confronted the issue of what it means to be conscious or, in other words, the nature of consciousness as self-referentiality. Research has tended to focus on the percept. Therefore, explaining the non-detachability of parts in subjective experience risks becoming a sort of impossible achievement, similar to that of Baron Munchausen, who succeeds in escaping unharmed from this quicksand by pulling himself out by his hair. This paper addresses how to analyze seeing as an undivided whole by discussing several basic dimensions of phenomenal consciousness on an experimental basis and suggesting an alternative way of escaping this quicksand. This mind-set reversal also sheds light on the organization and dependence relationships between phenomenology, psychophysics, and neuroscience.

## 1. Introduction: Seeing

The characteristics and behavior of visual experience over the last hundred years have received considerable attention, even if they have been analyzed and explained using an increasingly wide range of theories and models. Different approaches have often coexisted, albeit with alternating authority, such as ecological [1], inferentialist [2,3], autopoietic [4,5], constructivist [6], computational [7,8], representationalist [9], integration information theory (ITT) [10], global neuronal space theory [11], predictive coding theory [12], embodied approach [13,14,15], embodied constructivist theory [4,5], temporospatial theory of consciousness [16,17,18], signal detection theory (SDT), and so on. Briefly, we moved from the concept of a direct perception of the environment to the concept of an indirect perception based on probabilistic hypotheses and with an increasing role of computation models. This abundance of theories and approaches indicates both the richness and still indefinite foundations of psychological vision research. They have been successful in achieving a correlation between physical stimulation, brain states, and conscious visual contents, but none of them have been able to explain what consciousness (or subjectivity) is directly. Understanding the nature of subjective perception, therefore, remains a thorny issue in vision science and a battle that has not yet been won. In fact, no matter how many extraordinary results have been achieved in different fields of research, subjectivity evades the psychophysical and neuroscientific analyses conducted in third-person accounts for the simple reason that their methodologies delete qualities, which leaves them necessarily grounded in physics. As Libet puts it, there is no guarantee that the phenomenon of awareness and its correlated phenomena can be explained in terms of current physical laws or by our knowledge of nerve cell activity. It is a fact that subjective experience is accessible only to the subject having that experience [19]. Externally observable physical events and internal psychic events are categorically independent, and neither of them can be limited to or even described by the other [19]. To provide a thorough scientific explanation of subjectivity, what is missing is an understanding of exactly how consciousness—from the primary process onwards—works inwardly. This is because it is apparently impossible to separate the percept from the self-referentiality implicit in awareness.

On the other hand, phenomenology as a philosophical theory and Gestalt and Experimental Phenomenology as sciences have addressed the issue of subjectivity in perception directly, even if their conclusions vary considerably. Philosophers have developed a broad range of analyses on the subjective awareness of the world, both at a precategorical [20,21,22] and categorical level [22,23]. However, philosophical theory and its vocabulary are difficult and sometimes clumsy. This has generally dissuaded people working in science from making an effort to enter a scary and apparently inextricable jungle of acts, contents, and objects [22,24,25]. Despite this, I wish to argue that the above distinction is the foundation for any experimental approach to consciousness and awareness in the perception of a phenomenon. In fact, scientists with a phenomenological attitude from Gestalt psychology onwards have mainly been interested in the percept and its behavior (the phenomenal object), with a specific focus on vision. They have not focused on the act dimension of seeing. As far as the contents are concerned, i.e., what fills-in a visual presentation, even if they are distinguishable and identifiable qualities (such as the spatial, colored, and smooth textured appearance of a surface), they are not detachable parts of the object [the colored surface being seen] they belong to [21]. To visually understand the distinction between the acts, objects, and contents of a presentation, consider, for example, the Hering illusion. Here, drawings are physical stimuli, the perceived deformed shape is the object, the curved perceived lines are the content, and seeing is the act (see also below, Section 5).

Over the years, the original Gestalt attitude has merged with various cognitive and neuroscientific approaches [26,27,28,29], and one has the impression that it has been forced to resort to a kind of naturalization [30,31] to survive. Fortunately, phenomenology has never been targeted as scientific Dadaism, as Einstein’s theory of relativity has [32]. However, the dismissive attitude of many scientists in the field of vision science remains.

The key argument for this lies in the supposed inability of phenomenology to explain its results from the point of view of scientific “credentials”. For example, the notion of cause and effect, the asymmetry between explanans and explanandum, and a refusal to ground in and limit research to physics [33]. A psychological science based on the analysis of phenomena as they subjectively appear, in fact, seems somehow idiosyncratic compared to psychophysics and neuroscience for several reasons:

- First, phenomenological explanations need to exclude any reference to the classic idea of asymmetry (physical energy transmitted from one object to another, linear before-after of objects, etc.) [34,35].
- Second, the relationship between explanans (the necessary and sufficient conditions ruled by the laws of organization) and explanandum (the appearance within perception) simultaneously bond together in the phenomenon, making the task of detaching them extremely difficult.
- Third, causality in phenomenology is not a logical construct but a perceived phenomenon, even when it is endowed with expressive force [36,37,38].

However, we should not forget Koffka’s bold statement that, strictly speaking, unequivocal sensation exists only for the psychologist and is a product of the laboratory [39].

Building on the theoretical and methodological approach of Gestalt psychology, experimental phenomenology has obtained a further series of scientific results in vision science, including in amodality [40,41,42,43], light perception [44], figurality [43,45,46,47]; so-called perceptual illusions [48,49] see Pinna [50], color [48], pictorial space [51], and subjective time [52]. Even while resisting naturalization, these analyses based on subjective experience have never directly approached the issue of what it means to be subjectively aware during perception either or how to explain it (the Nagel [53] question). The focus has generally remained on the analysis of the perceived objects. So, while being crucial to phenomenological research, the nature of subjectivity remains a “stone guest” for several understandable reasons, which include:

- The difficulty of analyzing and testing the object of research (i.e., the percept and the varieties of its modes of appearance) in the brief experience of actual perception is separate from the unavoidable and simultaneous dimension of the subject’s awareness (the act, for example of “seeing”).
- The fact is that the unity and simultaneity of the momentary experience are only sub-whole in a more comprehensive entirety of experience (the psychic flow).
- Finally, a continued lack of knowledge regarding the nature of psychic energy. However, as Volkelt observed a long time ago, this is precisely the task of a psychology of consciousness [54].

These distinctions are also needed because scientists frequently use subjective awareness (the specious present) and consciousness (a larger continuous process) as synonyms, and sometimes the term awareness is used to refer to sensorial awareness, i.e., being sensitive to physical stimuli, which complicates the issue still further. It is also worth noting that in science, even the contemporary debate on the variations and gradations of consciousness has been developed mainly within the field of cognitive neurosciences, and physics has weighed in on the debate, too, given its role as the basepoint for bottom-up emergence. To achieve a thorough theory of consciousness, getting rid of this first step is necessary but not easy. In other words, it seems that, as Varela put it, “we can see the path, but nobody is on it” [55,56].

While a lot of information has been acquired on the qualitative matter of visual appearances (figurality, color, transparency, light, remoteness, etc.) and more recently on their intrinsic cross-modality, apparently, we still do not have a clue how to deal with or scientifically explain the nature of the subjective dimensions of awareness in perception. These dimensions, however, are precisely those that allow appearances to emerge and fade away. To a certain extent, even phenomenological experimental research can proceed by avoiding the thorny issue of what awareness in perception means, even if it exists at all. Ultimately, awareness is a fact of experience that does not need to be questioned unless it is seen as a permanent deception by a wicked god. We can, therefore, test, measure, and validate the behavior of participants engaged in certain perceptual tasks in a laboratory and render their responses in a first-person account, which provides a sort of inter-subjective objectivity, measured by a psychometric scale and supported and validated by statistical analysis [57]. Obviously, we cannot pretend that each subject will perceive appearances in exactly the same way, given that subjective variability is typical for biological phenomena, and the stimuli (appearances) observed are qualitative and, therefore, usually more complex than the distal stimuli from which they originate in the physical realm. The definition of qualitative stimuli escapes exact quantification [58]. So far, so good. However, as it seems impossible to give an explicit explanation of its grounding dimension (subjective awareness), phenomenology is required to merge with other scientific approaches, such as psychophysics and neurophysiology, whose grounds are manifest and explainable in terms of physics (for the use of psychophysics see [59]; for the scope and limits of neuroscientific analyses of consciousness [19,60]). In recent years, this claim has been raised by approaches such as neurophenomenology [56,61], micro-phenomenology [62,63,64], and front-loaded phenomenology [65,66]. So, we can say that in vision science, experimental phenomenology has limited itself to the descriptive and non-explanatory role attributed to it by other scientific approaches and methodologies. In short, it has accepted that it must play a supporting role.

It may appear somewhat odd, though, given its theoretical roots and scientific methodology, that experimental phenomenology has not explicitly tackled the issue of the nature of what it means to be subjectively aware, or more specifically, the nature of self-referentiality in the awareness of a visual phenomenon, which it verifies in the first-person. Questions about self-awareness, the boundaries of consciousness, and which methodology should be applied to address it have often been delegated to studies on meditation and eventually related to neurophysiology [67,68,69,70].

In fact, several clues regarding how to address the issue of self-referentiality exist already, both in the systematic and experimental research of phenomenological origins. What emerges from the original scientific approach is the concept of consciousness as simultaneous, ongoing, multiple, multifarious, phenomenal continua deployed in subjective space–time, which are the conditions of its existence. The macro and micro-structure of these continua, i.e., the temporal group-units, interwoven spatial positions, boundaries, and filling-in of its contents and objects (see below, Section 7), can be verified experimentally. Unlike other contemporary theories of consciousness, the approach is entirely qualitative [71]. Our future goal is to identify the dimensions, the primitives of these continua, their internal relationships, and the laws governing the process as a first step towards developing a notation and the psychometrics of the individual structural qualitative components.

My contribution discusses awareness from the experimental phenomenological viewpoint, and it is divided into the following sections:

- A brief explanation of the phenomenological standpoint in perception is provided (Section 2).
- The meaning of subjective in seeing and how it is experimentally addressed is specified (Section 3).
- The factors on which awareness is grounded are identified (Section 4, Section 5 and Section 6).
- The temporal scanning of awareness is presented and discussed (Section 7).
- The role of place in awareness is discussed (Section 8).
- Preliminary steps on the resulting geometry of seeing are presented (Section 9).
- The complexity of the phenomenal continuum in awareness is pointed out (Section 10).
- Conclusions and examples for a possibly fruitful collaboration between experimental phenomenology and neuroscience are provided (Section 11).

In this study, I shall limit myself to the basic structure of actual awareness. I am also aware that, as Wertheimer observed some time ago, any attempt to explain phenomenological issues in a short essay is extremely difficult because of the terms used, such as parts, whole, intrinsic determinations, and, I add, act, content, and object. This terminology has been the topic of endless discussions in which every scholar has their own interpretation [72]. Consequently, in my analysis, I will rely on experimental examples. To reduce the complexity, I have also chosen to focus mainly, but not entirely, on well-known phenomena and their experimental testing (see also Appendix A Table A3). What I argue here, in opposition to the main view [73], is that consciousness is entirely tractable and verifiable in its own terms. However, a scientific mind-set reversal is unavoidable. The main aim of this study is to promote new research on the neural correlates of the phenomena I am going to discuss without collapsing consciousness on neurophysiology.

## 2. A Brief Exposition of the Phenomenological Standpoint in Perception

Leaving aside all the painstaking technicalities of philosophical theory, we can simplify systematic research as follows. When defining what a psychic phenomenon (Vorstellung) is (for example, seeing), Brentano [20,74,75,76] distinguishes the threefold dimensions of the act (the presentation proper, a psychic process), content, and object as internally related parts, none of which can be detached from the others. It is worth noting that presentation refers both to subjective perceiving and imagining, which shows the wholly internal nature of the processes deployed in consciousness. The content qualities of a presentation, negatively described by Ebbinghaus [77] as non-vivid, immaterial, unstable, and without identifiable characteristics, have since been identified positively in terms of their plasticity, inner temporality, and spatial localization, qualitative and cross-modal contents, and subject to scrutiny by Gestalt Psychology and Experimental Phenomenology. So, contrary to what is now almost taken for granted, the original idea of presentations was as perceptual and not stimulus-based representations, whether they were conceived as products of information recovery and sensory completion, model representations [78], or estimating properties of “objective reality” with direct access to statistics represented in the mathematics of estimation [2,59,79,80,81,82]. The fact that the physics of the perceived environment does not follow Newtonian laws has long since been demonstrated (see, for example, [83,84]). Specifically, in perception, every act of seeing must inherently have a seen object [85], whether it is just a (φ) movement, and be aware of seeing, hence its indisputable evidence. For example, seeing a color implies the awareness of perceiving a spatial quality (a surface as part of the visual space), which cannot be detached from the textured quality (the perceived color of the surface) [20].

This holds for any variety of surface and color textures and includes these so-called anomalies:

- Surfaces are delimited by “illusory” contours, where brightness enhancement and contours are perceived without a luminance or color change across the contour [42,43].
- Surfaces of perceptual transparency depend on the perceptual spatial organization of differently colored areas [86,87,88,89].
- Surfaces with color spreading where a bright “illusory” disk appears superimposed upon the radial lines that complete amodally behind the disk [90,91,92,93,94].
- Surfaces in the watercolor effect where a colored line flanking a darker border will appear to assimilate its color onto the enclosed white area [47,49,95].

Addressing the above-mentioned phenomena as “illusory” is still a litany that is difficult to eliminate from scientific terminology. Over the years, for each of these so-called illusory surfaces, a cognitive, psychophysical, and/or neurophysiological explanation has been given (see, for example, [96,97,98,99,100]), but the value of their phenomenology, even if highly detailed, has remained complementary and limited to a descriptive and demonstrative task. So, while the content and objectual dimensions of perceiving have often been investigated (for example, the non-detachability of visual space and visual objects was demonstrated relatively early [101,102]), it is demonstrated that one can see movement without seeing an object that moves (φ), the nature of the act dimension and self-reference in seeing have so far resisted experimental research. In fact, this seems to be a dimension that is somewhat difficult to identify and extrapolate, with the presentation being the brief unfolding process of a unitary whole. This feat is again reminiscent of Baron Münchausen.

### Regarding the Task of Experimentally Developing Subjective Perception

Wertheimer’s groundbreaking research points to the internal nature of the perceptual objects (Gestalten) as processes and the role of their part-processes (contents) [103,104]. In so doing, in science, visual objects lose their usually unquestioned status of independent physical existence. Over the years, research has focused on the objectual or content-laden aspect of perception (i.e., “what I see” as opposed to “I am aware of seeing”). Think of the case of blindsight, when a stimulus is unconsciously perceived, but it is not present at the level of consciousness. So, as mentioned, what remains shadowy in the phenomenological approach and a source of limitation is the nature of the act component, i.e., the awareness we simultaneously have of the object we are perceiving. The role of this component can be shown easily through simple drawings such as Michotte’s parallelepiped. See Supplemental Materials, Figure S1 [105,106], a visually truncated pyramid with a transparent, strongly voluminous appearance that is shaped into the continuity of seeing; the more famous Necker cube [107]; or Escher’s prints [108,109]. In these examples, a change in the way of seeing alters the object, its spatial position, and its three-dimensionality. If you draw a black rectangle with a smaller white one in the middle of it, the white rectangle is seen to be either foregrounded against the black surface (a stratification of surfaces) or behind the black surface (such as a slit in a void background). If seen as foregrounded, the rectangle appears smaller and less clear cut, whereas, if it is seen as backgrounded, the rectangle appears larger and clearer cut (the example is Benussi’s. See Supplemental Materials, Figure S2). These different appearances have been explained as the outcome of assimilative and connective factors. However, they cannot be attributed only to the content/objectual parts of the phenomenon (i.e., the white and black surfaces with a qualitative depth index), as the role of both field and subjectivity apply to the explanation (see below, Section 4 and Section 5). Recently, neural correlates of bi-stable figures have been found [110], and multi-stable perceptions have been explained in Bayesian terms [111], but they neither apply nor explain the nature of the subjective factors working in awareness (see [112]; a discussion of perceptual organization and consciousness can be found in [113]).

It is worth noting that the awareness of a percept is not yet an egological and reflective consciousness that distinguishes a percept from a perceiving ego. Awareness is a non-detachable part of the perceptual whole (seeing), and this is precisely the difference between qualitative perceiving and physical stimuli representation. Brentano was very clear on this point. What is a given (es gibt) in a presentation is not (at least not yet) a subject detached from and representing something else. What is a given in the psychic present is an undivided whole, even of great complexity, of which self-reference is a non-detachable part (what has been called the stage of ante-predicative symmetry making, see [114]. This standpoint establishes one of the crucial issues in the definition of the subjective factors in phenomenal perception, namely, the identification and explanation of the nature of awareness with no reference to either lower or higher levels of introspective [62] or reflective [115] mediation. It is no coincidence that the contribution of phenomenology in neuroscientific research has been received and appreciated essentially in terms of providing ostensibly linguistic descriptions [116] to construe protocols and questionnaires [56,63,64]. As these situations imply language (i.e., cognitive functions), they are useful in preliminary neurophysiological investigations. In other words, once again, phenomenology only plays a supporting role. There is no doubt either that the relevance of subjective experience accounts should be considered on the same level as other types of evidence (even Titchener recognized introspection [117] and verbal reports), but the role of phenomenology in science is not limited to that (for a contribution on the relationship between perception and language description see [51]). The problem of explaining subjectivity remains grounded on the nature of non-detachable self-reference in perceiving. This difficulty increases because of the specific nature and duration of actual perceiving, which is a very brief, qualitative process that experimental research has only been able to model on the external metric of what has been variously defined as physical, mathematical, objective, chronological, and numerical time units. Mathematical time, however, is durationless, so it cannot represent the internal nature of the flow of consciousness, which proceeds on an orderly qualitative scale (see below, Section 5). Many aspects of the nature of presentations are, therefore, still far from being clearly understood and explained.

This is the battlefield, and I shall confine the present analysis to subjective awareness of the phenomena of vision, or what Kanizsa defined as the primary process and its group-unities, deriving from the elaboration of proximal stimuli [46,118]. See also [101,119]. An analysis of higher levels of consciousness, such as the larger succession of a continuous series of groups of simultaneous and multifarious phenomenal wholes in a sufficiently continuous temporal variation, including an analysis of secondary process actions, such as abstraction, categorization, assumptions, and inference [46,118] would require further research.

## 3. What Is Subjective in Seeing or “Give to the Subject What Belongs to the Subject”

All perceived phenomena are of a dynamic nature and take place over a duration [23,120,121]. There are no punctual percepts or static images, as some may argue [122]. Just think of the sensational motion illusion examples given by Kitaoka [123], Ouchi [124], and Pinna & Spillmann [125]; see also Pinna [126]. One can experience events that are stationary and stable, dynamic and unstable, or continuous or discontinuous, but strictly speaking, no discrete atomic states detached from the continuous subjective flow of consciousness can be experienced (Metzger offers a useful categorization of events [127]; see also [128,129]). Naturally, the behavior of the subjective factors in shaping appearances is particularly enhanced in the phenomena of so-called apparent movements. Apparent movements include a plurivocal series of perceptual events [130], such as movement without an object (φ) and configurational movement (β) [131] that, by no coincidence, led to the birth of Gestalt psychology (see below, Section 5). Stereokinetic [132] (see below Section 5), stroboscopic [133], and Ternus [134] movements are particularly informative, as they show:

- The presence and role of both empirical and internal factors of awareness.
- The phases of an actual seeing experience.
- The subjective space–time continuum is a condition for the existence of phenomena in awareness.

Note that subjective time and space interact and are mutually non-detachable in seeing. It has also been clarified that, for example, the time interval between successive stimuli influences the perceived spatial distance (Tau effect) [135], and the elapsed time between sensory stimuli is modified by their sequential presentation at different localizations (Kappa effect) [135,136,137,138,139]. These studies, which show the reciprocal non-independence of subjective space and time, also highlight their ontological dependence on the perceiving subject, which is the origin of their deployment: no subjectivity, no space–time awareness. The nature of subjective space–time (and its internal procedural rule) would remain a purely philosophical (old Kantian) question if it did not regard the ineliminable dimension of subjectivity in the awareness of a percept that is required to achieve the status of explicative science for experimental phenomenology. Unfortunately, in vision science, the subjective factors that condition the formation of a percept are often considered to be essentially rooted in the influence of (mental) past experience, which favors regression ad infinitum and, in my view, only pushes the issue further into the background. Adopting or not adopting this perspective condition and/or preventing the application of scientific research to the nature of the subjective factors intrinsic to perception as structural factors. It is, in fact, commonly used to account for patterns formed according to past experience ([96,138,140,141]; criticism in Kanhizsa [142,143]), but much less to investigate empirical and internal factors structuring perception at a primary level, that is too easily referred to as familiarity [144]. Nevertheless, to test, validate, and explain the nature and role of these factors, thereby allowing (or explaining) the existence of the perceptual objects, requires experimental and systematic analyses to be considered and implemented. So, in the following section, we will discuss in detail the nature of these subjective factors and their behavior in shaping a visual object.

Here below, I refer to a few experiments highlighting particularly conspicuous factors regarding homogeneity in shape transformation (in subjective time), the identity of shapes (in apparent motion), and the subjective temporal conditions (in subjective space localization). Most of the experiments are classics in vision science, and for those less well-known ones, more details have been added in the text and/or Appendix A. To avoid the misunderstandings that frequently occur between the correlated sciences of psychophysics, neurophysiology, and experimental phenomenology, it is advisable to make an immediate distinction between the nature of the stimuli and the type of variables considered in the different scientific domains. In experimental phenomenology, stimuli (the observables) are not physical, such as the features, cues, edges, etc., of vision science (such as frequencies in acoustics). They are phenomena such as the appearance of color, shape, and light and their characteristics [48]. The latter has sometimes been considered vague, if not infamous, because they are sensitive to context (for probabilistic feature models, see [145]). Obviously, no one denies the presence or weight of the physical stimuli and neural correlates underlying the phenomena. However, the latter are not limited to the former because they are governed by different laws, specifically by qualitative laws, as we have just discussed. Furthermore, unlike other psychological sciences, experimental phenomenology considers only phenomenological variables. Independent and dependent variables that belong to the same (phenomenological) domain are simultaneously observable in terms of present awareness and measured as such [119]. Consequently, an explanation of the phenomena must also be given at this level.

## 4. How to Identify the Factors of Awareness

Phenomenological analyses of perception provide a huge amount of evidence to show that what we perceive consists essentially of relational phenomena that allow temporal dislocations [52,146] and spatial deformations (and even their shadows in 3D, [144,147]). This includes the geometry of the subjective, anisotropic visual space [51,57,118,119,148,149,150,151,152] and the microgenesis and dynamics of subjective time in the specious present [121,153,154,155]. For example, given a very brief succession of high and low sounds (physically, stimuli such as 1.760 Hz, 82.4 Hz, and 1568 Hz, in succession for 100 ms each), a temporal dislocation is perceived. So, what is heard is the rapid succession of two high sounds, followed by a low one ([52] and below). In other words, the forward axis of the physical time of the stimuli and that of the subjective time of the phenomena in awareness do not always follow the same order. Phenomenologically, the reason is simple. High notes group together, expelling the lower ones, due to the qualitative grouping of similar parts (in tonal highness) in awareness, which is an example of the roles of similarity and tendency to good shape in acoustic perception. The categories of before–after, as applied to physical events, do not rigidly hold for perception because of the non-independence of the parts of the whole, and the whole is governed by the qualitative principles of organization [see also the Window effect ([129,156] and below). In other words, temporal Gestalten are produced in the specious present. The phenomenon of dislocation also occurs in the visual field once spatial influences have been experimentally eliminated [157].

In the awareness of a percept (object), different factors can be identified: in addition to (1) the natural factors described by Gestalt psychology (following the principles of figural organization) (p. 352ff, [158]), (2) empirical factors that contribute to the process of structuring the perceived objects can also be identified. Musatti [128,159] speaks about them in terms of assimilative factors and field factors producing modifications, transformations, and enrichment of the phenomenon, an analysis that, unfortunately, has been frequently misunderstood in terms of individual past experiences. On the other hand, the nature of the (3) internal factors of awareness have been investigated by Benussi, even if he, unfortunately, defines them as “inner determinants of the act” (i.e., the internal factors of the process), a definition that only a true Meinongian would fully understand. The characteristics and role of empirical and internal factors have also been experimentally considered and shown to work together simultaneously as conditions of appearance for the objects of seeing [160,161]. In the following sections, let us analyze the role and behavior of factors (2) and (3).

## 5. Empirical Factors: Field Organization

One of the most conspicuous examples of the role and behavior of empirical factors (2) in shaping a visual object is given by stereokinetic phenomena that visually mesmerize anyone who experiences Duchamp’s rotoreliefs at the Guggenheim Museum. I have discussed these phenomena in detail elsewhere [162]. See also [163]. Neural correlates of the phenomenon have also been found, which provide valuable scientific results but neither apply nor explain what qualitatively appears in awareness when we see them. Interestingly, the phenomenon is seen with lesser or greater vividness according to different subjective attitudes, and obviously, unless you have stereoscopic vision, you can probably only figurate it when “thinking”. The key points of the phenomenon can be summarized using an example that involves rotating a black cardboard disk with curved white lines drawn on it. See Supplemental Materials, Figure S3 [159,164]. When we see this, we progressively perceive:

- A flat rotating ellipse with a constant shape on a single plane.
- An elastic ring or self-distorting figure on the plane that is stably oriented in space.
- A rigid circular disk or constant figure that tilts back and forth in space while rotating.
- Finally, the disc disappears, and the ellipse becomes a rigid ellipsoid (egg-shaped object), tilted in depth and with a definite length [159,160,164].

In terms of awareness, the continuity of the process is nevertheless characterized by a series of shifts in a plurality of sub-objects (ellipse, ring, disk), which act as non-independent parts (contents) of the whole phenomenon and tend to create a better, final, stable shape requiring no further improvement (Pregnänz as a process tending towards stability in the perceptual field [112,165]). The phenomenon has been explained using various hypotheses (rigidity assumption [128,159,166]), likelihood principle, [141], and object hypothesis [167], but the best hypothesis that accounts for the different transformations is that of velocity difference minimization (VDM) [164]; for the perception of velocity see [168,169]. What makes stereokinetic phenomena interesting is the appearance of the rotating ellipse (or other similar forms), which are created by a continuous visual process that gives rise to more than one outcome (the ellipse and the ring being non-detachable parts of the final whole) [164]. This reveals the potentially hierarchical internal over-construction structure [170] of phenomenal objects in seeing, as they are categorically independent of physical stimuli (which remain unchanged during the whole process) and existentially dependent on their bearer (the act of seeing, its subjective timing, subjective localization, and subjective factors). Finally, stereokinetic phenomena demonstrate the internal emergence of form in awareness. There is no need to refer to neural bottom-up and/or cognitive processes [171] to explain the emergence of form in awareness! Kanizsa [143] has already warned against what he calls the error of expectancy. The final stabilized object (Gestalt) is shown to be a complex construction due to the successive modification of the parts of the moving complex to achieve greater homogeneity of the whole. Musatti upholds that the general principle of homogeneity governs the whole perception and that all the other principles previously analyzed by Wertheimer [103,104] lead back to this factor, including the concept of Prägnanz [132].

The assimilative factor explains how perceptual data in awareness are experienced as becoming a whole of objectual qualities (contents) and gives some information on the nature of the non-detachable visible space in which they are localized (see Section 8). More specifically:

- The stereokinetic phenomenon is perceived in awareness as a glowing and spacious object if compared with two-dimensional surfaces such as a drawn ellipse.
- All the parts are perceived as moving together.
- Information is given on the location (and direction) of the phenomena deploying a change during their deployment.
- The presence of empirical factors is wired into the phenomenal field [112].

The most important aspect of Musatti’s analysis of stereokinetic phenomena, however, concerns how the internal relations of the contiguous non-independent parts/contents in the process (ellipse, ring, disc) form a continuous whole through a serial order of spatial positions. This has led to an analysis of the transition between rigidity and non-rigidity of objects in terms of priors [172], studies on how neural circuits are engaged when the human brain combines different information sources for obtaining a 3D visual perception [173], and studies testing the relevance of the white-matter pathway for “learning” the temporal structure of event streams [174]. All of which, however relevant, do not explain the phenomena as they are subjectively experienced. Assumptions, methodologies, observables, and variables in the different research fields cannot be considered equivalent, even if the results can be correlated.

## 6. Internal Factors: Subjective Timing

Let us now consider the role and the behavior of the (3) internal factors temporally shaping a percept. Phenomena appear and are experienced in subjective time (the so-called flow of consciousness). The experimental research on this matter is still considerably less than on other vision science issues, such as shape, color, depth, light, texture, symmetry, shade, contour, and so on, even if it is progressively gaining attention. In recent decades, the topic has mainly been discussed from a cognitive [175,176,177,178,179] or neurophysiological perspective [180,181,182,183,184,185,186,187,188]. See also the semantic approach by Callender [189]. Contrary to this trend, research on subjective time was incredibly rich between the 19th and 20th centuries, gained theoretical and experimental relevance in the thirties and before [190,191,192,193], and was successively expanded by Fraisse [194,195] and Vicario [52,196]. This research foreshadowed many questions that are still at stake. The most comprehensive discussion of the experimental research and new results in this area is offered by Vicario [129]. Other miscellaneous contributions, ranging from philosophy to neuroscience, have been collected by Arstila & Lloyd [197], where discussions and comparisons with the phenomenological approach appear in contributions by Dainton [198] and Pöppell & Bao [199].

In this paper, however, I have chosen to focus on two well-known phenomena that emphasize the behavior of internal factors in awareness. Stroboscopic movement [133,145] shows the co-presence of two phenomenal types of movement: the first (S-movement) occurs through a series of stroboscopic images; the second (S-movement) occurs because of the perception of spatial Gestalten and is determined by the movement of the different individual parts of the figure. This experiment highlights some crucial issues of what occurs in awareness. The first concerns the question of whether the spatial forms produced by the temporal phases of a very brief movement are perceived or not. The second regards the presence of subjective settings, such as the so-called analytic (A-reaction) or the synthetic (Gestalt-reaction), attitudes that can modify (although not delete) the asensorial nature of the phenomena [200]. The existence of at least two subjective attitudes in the perception of phenomena has been successively confirmed in the perception and evaluation of temporal strings [201]. Not surprisingly, the debate on stroboscopic movement (instigated by a variation of the Müller–Lyer figure, see [202], became a source of controversy between Berlin (Koffka and Wertheimer) [203] and Graz [137]). However, the different assumptions regarding the self-organization of percepts (objects) and the weight of the internal factors structuring phenomenal awareness (the experimental scanning of the act) remained the same (See also [204,205,206]).

Another well-known phenomenon that is also extremely informative about the behavior of the (3) internal factors is the gamma effect [203,207,208,209]. This phenomenon refers to the perception of a movement that expands from the center to the periphery of a surface that is wholly and instantaneously (physical stimuli of about 100 ms or even less) illuminated or obscured. In the first case, the illumination is perceived to expand in a centrifugal movement from the center of the surface to its periphery, while in the second case, the illumination is perceived to move in the opposite centripetal direction, contracting from the periphery to the center. Subjectively, observers report having the impression of an “instantaneous” flash, even if they also describe perceiving “two phases” of a movement (expansion and contraction). Similar experiments, whose task is to evaluate whether a certain line that is shortened or lengthened is longer or shorter than a stationary line, lead to the same apparently paradoxical result of conflicting perceived timing [210,211,212] and to the same conclusion that the phenomenal perception of instantaneity corresponds to different physical instants.

Stroboscopic, gamma, and many other phenomena help to deepen our understanding of the microstructure of awareness in several aspects, including the perceived length of its permanence/durability [213,214], its relationship with the content and object, its vividness, and its internal factors as conditions for the possibility and existence of the phenomenon in consciousness. What we learn is that, even when non-detachable parts are deployed in a presentation, one has to distinguish the time of the act (the process), the time of the object (the phenomenon), and the time of the content (an old issue among Meinongians, see [24]). From this perspective, it seems natural to conclude that the research conducted on subjective time is experimental (hence verifiable) phenomenological research on one existential condition of experience, and one could add on what reality is in awareness, which may be a distinctly indigestible conclusion for many vision scientists.

## 7. Scanning Awareness

After considering the behavior of the (3) internal factors, the question is how to verify the rules of their temporal development in awareness. Our experience is that the flow of consciousness (even when subjectively related) inexorably flows and is usually experienced without gaps, even in cases of psychological waiting Erwartung, [154], i.e., perceiving an (empty) interval or anticipating other aspects of experience that may potentially appear (from a psychophysical and neurophysiological viewpoint; see [215]. See also [216]. From a philosophical viewpoint, see [217]). In this respect, experiences of awareness without objects, such as acts of evidence, doubt, assertion, or denial (identified in induced hypnotic sleep by Benussi [161]), are interesting. In fact, even more strikingly than what occurs with the (φ) phenomenon or the gamma effect discussed above, in these experiences, the act dimension prevails. To break through the continuity of the flow and explain how it works from within without resorting to external psychophysical and neural information and models (Northoff et al., 2020 [26,218,219]) is obviously an engaging task. The issue of addressing its potential psychometrics is based on the purely qualitative differences perceived in awareness (“more or less short”, “more or less long”). Something similar, however, has been considered for color appearances, which has given rise to the Natural Color System (NCS) notation, where color is identified and measured, for example, as “more or less red” or “more or less yellow” [220]. For some dimensions of subjective time, the question may be more complex because, in color perception, the focus of the notation is still on the object to be evaluated. Nevertheless, no matter how difficult it is to accomplish, in principle, a qualitative notation such as NCS should be obtained for temporal durations as tentatively achieved by Benussi ([154] and Appendix A, Table A1). Benussi’s salient duration findings identify wider and narrower dimensions of awareness and place the limit of the “now” (the specious present) at the end of verified very long durations (of about 3 s) when, according to his theory, the vivid “presence” of a phenomenon in awareness shifts to “mental” mode (i.e., from the perceptual primary process to mental cognition) [46,144]. Benussi’s durations have been confirmed by Calabresi [192] and more recently by D’Angiulli and Reeves [221], with some slight variations (see Appendix A, Table A2). In addition to these confirmed durations, D’Angiulli and Reeves have also verified Bonaventura and Calabresi’s conception of vividness (a concept that is still indeterminate and subject to widely different interpretations in the scientific literature, see [222]) as the intensive gradient and variation of present awareness and its potential correlation to the brain’s intrinsic cycle of 1 s. The Kantian idea that every object of experience possesses a degree and continuous intensive gradient of reality, which can be evaluated only in terms of continuous increase or decrease, should also be taken into consideration here [223,224]. The main point to clarify, however, is that the quality and degree of the gradient depend on specific durations (i.e., from the act dimension) [225]. In fact, by comparing the results, we can identify the actual full, clear, and vivid presence of a phenomenon in awareness in about 1000 ms, while a larger extension of awareness (the specious present or “now” as a temporal field) ranges from about 100 ms to 4 s. Phenomenologically, below 100 ms one can perceive something as qualitatively instantaneous (although not an instant, as previously discussed); above 100 ms one perceives durations. The durations identified as bounding the “now” are influenced by the subjective attitudes (analytic or synthetic), attention [34,226,227], emotions and cross-modality [228], content complexity, the intensity and quality of the limiting sensations, the homogeneity of the assimilative factors, the intuition of the proximity of past and future moments [197], etc. Consequently, even indeterminate durations (of about 1000 ms) cannot be rigidly and numerically counted. The question of whether we can numerically identify psychic temporal “moments” [229] is fallacious because 60 ms or even durations of 100/150 ms (putatively mapped on instants of objective time) can exist only in relation to a larger whole of actual awareness. They cannot be detached and classified as “numerical units” of time that can be added to one another to identify the breadth of the now. If required, I would define them as “qualitative (non-rigid) group units”. A notation of these and other identifiable durations in a system needs to be studied and experimentally tested and developed.

An interesting correlation between the above-mentioned phenomenological research and neurophysiological processes can be found in Libet [19], who has shown that subjective is not identical to neural timing but is backdated to the neural event (a delay of 500 ms) (a fact that bears on free will as well. See also [230]). In short, the “now” in awareness may not be synchronic with the now of the stimulus reception. Libet recognizes that even a thorough knowledge of neural activity would not reveal anything about the subjective experiences they may induce. In fact, no neural mechanism has been found that can be considered to mediate the subjective. As the transformation of neural patterns in subjective presentations develops within the mental level that emerges (I cannot help but think that Brentano has finally been avenged!). So, even if the non-sustainability of a reductionistic theory for consciousness has been experimentally established [19,231] for a neurophysiologist, the nature of its emergence has yet to be explained. This is what experimental phenomenology does by analyzing and experimentally verifying the nature of the existential conditions for its internal construction and deployment. Subjective durations do not correspond to states of neural duration in the brain.

A second neurophysiological correlation for some of the above-mentioned subjectively experienced durations has been found by Pöppell. For example, 30 ms and 2/3 s have been defined as temporal windows for integrating (physical) information [180,232]. Pöppell defines temporal states in the temporal domain of 30 to 60 ms as “elementary integration units”. It is worth noting that these neurological states, as opposed to subjective durations, are neither determined nor influenced by the contents that are processed. On the other hand, the so-called “anticipated moments”, that result from the neurophysiological analysis of attention and are related to the field of cognitive neuroscience [233], are attributes of incoming sensory stimulation. So, they are neither comparable to the concept of subjective group-units in awareness nor do they complement their subjective spatial parts. The brain as a “predictive organ” and consciousness as an anticipatory subjective process are not the same. In the same way, the hypothesis that the duration of the psychic present may be linked to the brain’s intrinsic cycle of about 1 s [221] shows a correlation, not an explanation of what phenomenally occurs in the flow of consciousness. Internal and external phenomena are independent of each other, and neither of them can be limited to the other. The same is true for studies on the different brain areas involved in temporal processing and the evaluation of time intervals below and above the 1 s range [26,181,218,219].

A third correlated neurophysiological analysis is given by Northoff’s proposal of Spatiotemporal Neuroscience (STN). Unlike Libet, and differently from causal theories of consciousness (such as Tononi’s IIT and Baars’ WGS), Northoff supports the existence of temporo-spatial dynamics common to neural and mental activity, which would also explain the construction of the mental and the characteristics of consciousness (such as self, time, and speed perception). Northoff’s hypothesis connects neuroscience to (contemporary) physics and maintains that the brain dynamics of space and time are a sort of “common currency” between neural and mental activity. According to this proposal, the degree of space–time integration on the neural level can predict the degree of consciousness (the self) and its temporal integration across different temporal points on the psychological level (however, on points in awareness, see below, Section 9).

Apparently, Northoff’s hypothesis comes close to the phenomenological perspective (Northoff himself makes explicit reference to the phenomenological tradition regarding mental space–time [17]). We agree on some aspects of his proposal, and precisely: (i) that consciousness requires spatiotemporal relational structures; (ii) that temporal dynamics and spatial location are mutually dependent; (iii) that both neural and mental dimensions are dynamic; (iv) that these relationships are part of the objects and events we perceive; (v) that being conscious of something is not a representation of physical stimuli; and, (vi) that it is mandatory to shift the focus of the research from stimulus-induced tasks or task-evoked activity to the spontaneous activity of the brain. However, some other aspects of his proposal are phenomenologically highly problematic. I am particularly puzzled by the disappearance of mental content. According to Northoff, the neural activity of the brain identifies physical space–time and mental space–time. No distinction is allowed between the quantitative dimensions of the physical–neurological activity of the brain and the qualitative dimensions of phenomenal awareness. From a phenomenological point of view, internal time and space are not a construction of brain activity but a categorial novelty; that is, they are different types of space–time, not merely different levels of the same space–time type. Phenomenological space–time is characterized by qualitative dimensions: you may consider the pervasive anisotropy of visual space and the subjective characteristics of psychic time. Moreover, in subjective temporal perception, one can identify different nested scales (consider, for example, the difference between indeterminate and extended durations of the psychic present or the multistability of several visual perceptions). The qualitative nature of subjective perception is the main assumption of phenomenology and its scientific outcomes (such as Gestalt psychology). As with other proposals coming from the neuroscience mainstream functional approach to the theory of consciousness [234,235], Northoff’s proposal also ignores qualities and reduces them to neural mechanisms of the brain [71]. What remains of the “mental” in Northoff’s approach is simply the word because the neural and the mental are both collapsed on quantities. Moreover, subjective space and time as conditions of existence of awareness have no room in Northoff’s theory [17].

By way of summary, the assumptions, methodologies, observables, and variables of the above-mentioned neurophysiological research are not equivalent to the analyses conducted in experimental phenomenology, although their results can be properly correlated. Knowing how perceptual phenomena and their physical and/or physiological causes are correlated has relevant outcomes for a variety of other sciences and technologies.

As to the experimental phenomenological perspective, the qualitative analysis of subjective time as an existential condition for a phenomenon to appear in awareness is complicated further by the fact that visual experiences unavoidably extend into subjective space. When taken separately, there seems to be an asymmetry between the (often assumed) linear order of time (derived from metrical time) and the placement juxtaposition of the experienced 3D space (above/below, right/left, front/back). What happens, in fact, is that in seeing, visual forms appear and are localized in phenomenal space along certain non-atomic durations, in which content may vary before assuming its final stable aspect (see above, Section 5). Wertheimer correctly speaks of the stationary character of perceived objects, where the qualities of the phenomena fill the space; they are internally localized in [21]. This gives rise to another unavoidable issue: the nature of an internal spatial geometry of awareness based on proper “elements”, which I am going to discuss in the next paragraphs.

## 8. Subjective Localizing

Where and how are the internal factors (2) and (3) localized in awareness? The procedure for identifying the visual dimensions of a phenomenal spatial continuum in the flow of consciousness starts from our experience of the environment, whether that is a simple colored surface, a thicker or narrower intertwining of branches, the way mountains seem flat when at a distance, and the voluminous appearance of clouds, fog, dunes, and water masses. In seeing, these formations can appear in different guises. For example, what in a starry vault appears to be a series of bright dimensional “points” of light or as relief drawings popping out in constellations can, in some latitudes, transform into bright voluminous 3D “balls” that look similar to lamps hanging from a chandelier (the experience can be so striking, one might even lift a hand to try to touch them). Nothing you know about the physical reality of stars and the atmosphere helps to explain how you qualitatively see these points or balls of light. To identify, classify, measure, and model the subjective appearance of visual dimensions, once again, we should follow Hering’s advice to start analyzing perceptual phenomena by refusing to mix them up with their physical or physiological causes or deriving from the latter any principle for their classification [85]. So, accurate observations of the subjective environment [101] may lead to the conception of a series of laboratory tests to verify the structural units of analysis for the visual field. For example, if something is perceived as a visual point, a dotted line, ball, or sphere, what is its location, and where should the boundary be placed between these different appearances? The complexity of this task is easy to understand given the multiple and multifarious visual configurations (often cross-modally experienced) that can naturally be perceived in a presentation. These percepts, similar to any other, lack immediate quantification and show a certain degree of ambiguity and expressivity [236].

The relationship between the perception of the localization of objects and the perception of the objects themselves is particularly interesting. In fact, the figural identity of objects is far weaker than the position component. Experiments show that in awareness, a shaped form moving forward in subjective space is not there at the start, but our awareness preserves its (past) position and constructs (or modifies) its initial content to the final localization (Gestalt position). Gottschaldt’ [237], Werner’ [238], and Kolers and Pomerantz’ [239] have all shown that contour is a relatively late aspect in the formation of objects, even if our visual system scans some of its characteristics from the start. Strong support for this idea has since been given by Kolers’ two-component model that is based on the distinction between two types of signals: the horizontal signal (H), which provides information on the localization of the stimulus (where), and the vertical signal (V), which provides figural information [what]. The H signals are processed more rapidly than the V ones; for example, whether the (φ) motion is seen at a greater physical distance than the (β) motion would depend completely on localization [240]. The underlying neural process of identification of visual objects is demonstrated in the seminal work of Milner and Goodale [117], which identifies two distinct visual systems, one for the what (ventral path) and one for the where (ventral pathway), which are processed simultaneously. This discovery, as is well known, has led to a few successive neurophysiological analyses. Similar to many other examples, excellent neuroscientific studies offer detailed information on the correlation between different kinds of observables (neural processes and phenomena). However, they are not the right tools to explain what occurs in the subjective experience of seeing. To do this, we need to remain at the phenomenological level.

## 9. What and Where in the Geometry of Seeing

Experimentally scanning the phenomenological spatial process in seeing reveals a perceptual hierarchy, including:

- A 3D Ganzfeld experience, i.e., 3D space (foggy, penetrable by sight, but spatially undetermined, such as thick fog [241,242]).
- Reasonably precise locations in space (where).
- (Usually) 3D objects (what), seen at first sight (immediately, globally).
- Other “parts” of former 3D objects.
- Surfaces, lines, and points are perceived equally as visual objects.

Studies on the three-dimensionality of objects and their parts are well known [243,244,245]. Much less attention, however, has been given to visual points, lines, and surfaces as geometrical units in seeing. Whatever kind of geometry and mathematics have been considered for vision, the models have always had a starting point in abstract entities and definitions. I have discussed how to analyze the main characteristics of the visual units or “elements” in the geometry of seeing in detail elsewhere [148,246]. So, here, I will simply summarize the basics by mentioning two correlated experimental studies.

Let us start with visual points.

In seeing, to classify a point, similar to any other visual object, one has to determine the kind of percept (what) and its position in visual space (where). This separate classification refers to the two different modes of being for a visual point (similar to the way colors appear as surface, film, or volume colors). Visual points, therefore, have specific characteristics. They are not dimensionless; they are localized, colored, often textured, and expressive (the points can appear good or bad, stable, unstable, and so on. See [36]). In short, they have width and thickness. All visible points have small surfaces or are small spheres, so they are not points in Euclid’s sense. Color and texture influence the appearance of a variety of visual points by modifying their spatial attitude (remoteness, invisible or visible line meetings, positions, etc.) in visual configurations.

Some of the characteristics of visual points have been experimentally tested by da Pos in three experiments (presented at SEQS 2013, CIMEC, Trento University [247]), but unfortunately, they have not been published. These experiments studied several characteristics and behaviors of visual points, including visual points as objects, to determine the size of a dot for it to appear as a point and not as a surface or line; the boundary (size threshold) beyond which a dot is perceived as an object, and when it is perceived as a point; the boundary between 3D dots and visual spheres. Here, I am allowed to briefly report the three experiments, with the hope that they will spark further studies on the topic.

In the first experiment, seven dots of the same achromatic colors (either white, medium grey, or else black) were shown on a monitor screen with two different local backgrounds (white and black). The four combinations were: 1—black background/white dots; 2—white background/black dots; 3—black background/grey dots; 4—white background/grey dots. The external surround was always white. The nine sizes of the dots for each background/point color combination spanned in three ranges (small range: 0.03–0.1–0.12–0.13–0.2–0.23–0.26–0.3–0.36 deg; medium range: 0.16–0.23–0.26–0.3–0.33–0.36–0.39–0.4–0.43 deg; large range: 0.16–0.2–0.23–0.3–0.36–0.43–0.49–0.56–0.63 deg). Therefore, the experiment was subdivided into three separate steps, performed in random order. In each experimental trial, seven dots of the same size, randomly distributed inside a virtual rectangle, were presented on the screen. The task was to observe the stimuli in free vision at 60 cm from the monitor and state whether they appeared similar to points (p) or small discs (d). Each participant performed a total of 108 different trials (three size ranges, nine sizes per range, four color combinations), in which the position of the dots changed from time to time by placing them a few millimeters away from the previous position at a random distance. The size of the dots was randomly changed trial by trial.

The results showed no statistical difference as a function of the background/dot color contrast; therefore, data for the four conditions were merged. The three thresholds separating the impression of the point from the impression of the disc were 0.22 deg for the dots belonging to the small range, 0.30 deg for the dots belonging to the medium range, and 0.35 deg for the dots belonging to the large range. They show that it is possible to distinguish the size of a perceptual point from the size of a perceptual object different from the point.

The second experiment aimed at finding the boundary (size threshold) beyond which a dot is perceived as a disk and when it is perceived as a point by the psychophysical method of limits. A single series of nine stimuli (seven dots each) was used (0.10, 0.16, 0.23, 0.30, 0.36, 0.43, 0.49, 0.56, and 0.63 deg each dot), once in ascending order and once in reverse order, each time starting from a different stimulus from the previous lap. The given task was to answer whether they saw a point or a small disk. Each participant performed 80 trials (ten ascending and ten descending runs, four color combinations). The results obtained with the statistical method of limits were congruent with those of the first experiment obtained with the statistical method of constant stimuli. On the one hand, background/dot color contrast did not produce statistically different results, and on the other hand, the dot of 0.30 deg SE 0.005 was the threshold separating perceived points from other objects.

The third experiment concerned 3D dots (stimuli were six series of seven small beads, 3, 4, and 6 mm each, three white and three black) and aimed at finding the boundary (volume threshold) between the visual dots and the visual spheres. Materials consisted of six gymnastic hoops, to which very thin, almost invisible nylon threads were tightened, and the seven beads in each series were tied at various distances, one from the other (from 2 to 4 cm). The beads were observed against a white for black beads and a black for white beads background and were illuminated at about 250 lux by a D65 source.

The given task was to answer whether the beads appeared similar to points or spheres in 72 trials, subdivided into 12 series as a function of the bead size and the bead/background contrast. A gray cardboard screen was used to hide the scene during the stimuli changes. The point/sphere threshold was found at about 0.21 deg for white spheres against a black background and about 0.24 deg for black spheres against a white background. Results confirmed, as expected that the white spheres appeared larger than the black ones, and the threshold for seeing a dot instead of a ball was lower for white spheres.

Although these results are tentative and need further research, they show that:

- Visual points are real perceptual objects.
- Visual points can be perceived in two different modalities: as objects and as locations in space.
- The boundary between a visual point and something else, such as a disc or a sphere, can be identified and measured (in the three tests, the thresholds ranged between 0.2 and 0.35 deg, depending on the test design).

What results from the analysis is that visual points have characteristics of their own; thus, the concept of an unextended (geometrical) point can be considered only a limit and a product of mental abstraction.

It would be interesting to design further experiments focusing on other aspects of visual points, such as different colors, textures, shapes, orders, expressive values, and cross-modalities; the contextual effects on these characteristics; and how the perception of a point as a location is characterized precisely in different contexts, for example as the result of a motor response. Because of the similarity between the visual field and visual arts, painting and drawing would be a mine of information for designing these experiments [248]. Up until now, only quasi-stable events (images) have been verified in the lab. So, further verification is required regarding the dynamic transformation of visual points into lines and discs in actual configurations. Finding the neural correlates of these perceptions would be deeply interesting.

### 9.1. Lines

The same complexity holds for visual lines, as there are only partial vision science studies regarding lines as rods (especially in haptic fields or frames). Visual line appearances are multifarious in the environment. Consider the intertwining of branches, the horizon line, blades of grass, leaf veins, stripes in animals, and so on. Visual lines are one-dimensional, as in drawings, but they also have a marginal second dimension. To be phenomenally visible, a line must have a certain width. So, to define a visual line, it is essential to verify:

- The what and the where.
- When the line ceases to appear as a line and becomes a surface or hole.
- If the line has a specific orientation.
- If color and texture influence its appearance.
- If the spatial attitude (curvature, shape position) is changed to a more complex visual configuration.
- If and how it is remote.
- If it is cross-modal (the cross-modality of lines is evident when complex musical pieces are transcribed into lines of different width, similar to Klee’s transcription of Bach’s Adagio. See [249])
- Finally, if it has an expressive value.

A recent series of (four) experiments has also sought to identify the visual line as a spatial element [250]. The stimuli adopted were [4] physical segments resembling Kandinsky’s style, produced through a complex procedure that makes them appear as natural as possible. Five variables were selected and tested to verify their role in the categorization and respective visual weight of the lines. These were:

- Type of line (straight or curved).
- Average thickness (five different levels of 0.5, 1.25, 3.1, 7.8, and 19.5 mm) to verify if and what was the visible boundary between the line and the surface; orientation (horizontal, vertical, or two 45-degree diagonals).
- Color (black RGB 0, 0, 0 and white RGB 255, 255, 255 in the first and third experiments; light blue RGB 133, 226, 236, dark blue RGB 10, 36, 82, brown RGB 67, 26, 6 and yellow RGB 255, 206, 14 in the second and fourth experiments). Isoluminant blue and brown were chosen from a perceptual viewpoint.

In the first two tasks (Experiments 1 and 2), the participants were asked to evaluate whether the stimuli were visual lines or surfaces.

In the following two tasks (Experiments 3 and 4), conducted with the Osgood semantic differential, the participants were asked to evaluate the stimuli according to the pairs of opposites presented on the screen.

The experiments gave the following results:

- Achromatic stimuli are more consistent than chromatic ones, and the thickness and type variables are the most important.
- The color variable is less important, and the orientation variable is generally not important at all.
- Straight lines are mainly evaluated as surfaces and perceived as cold, masculine, sharp, bound, flat, passive, static, acid, and geometrical.
- Curved lines are mainly evaluated as lines and categorized as warm, feminine, sweet, fluffy, dynamic, sensual, rounded, soft, and agitated.
- The boundary between visual lines and surfaces can be measured (in the experiment, it was between 0.5 and 19.5 mm).

The methods used in this study involved subjective first-person evaluations and a semantic differential scale, and both gave similar results. This is a good start, but other experiments need to be conducted, such as varying the thickness, orientation, and colors of the stimuli. In this case, too, only quasi-stable events (line images) have been verified in the lab. So, further verification is required regarding their dynamic transformation into surfaces during actual seeing. Also, in this case, finding out the neural correlates of these perceptions would be extremely interesting.

### 9.2. Surfaces

Visual surfaces are also multifarious, and several studies have verified their appearance, including glass [251], a transparent marble veil [252], painting [86], velvety skin [253], and so on. In fact, even a single line that crosses itself can make a visual surface stand out (Klee, Abstract Trio) [104]. Visual surfaces seem to have an inside and outside (perceived as negative and positive), different orientations, and deformations outwardly and inwardly [147], and they may appear rough (rocks), smooth (dunes), warm (a red textile), cold (metal), and so on. Visual surfaces are distributions of qualities and relations that fill the subjective space–time of a presentation [21,71,254]. Color, in addition to being non-detachable from surfaces, also determines their stratification and the stratification of surfaces goes hand in hand with their function as a dividing border. When surfaces are perceived to be on the same plane, their edge assumes the double function of being a contour closing one surface and a contour closing the other [48,255].

Given the similarity between phenomenal and pictorial space, the results of experiments measuring depth and surface attitude conducted by Koenderink and van Doorn are interesting [253,256]. In fact, they have identified several measurement methods for analyzing surfaces in pictorial space. These include the method of adjustment, in which the observer has to adjust a pointer to indicate the direction or curvature of a pictorial relief; the method of reproduction, where the observer has to reproduce a pictorial entity in terms of another one, potentially in a different pictorial space, in order to reproduce the depth difference as a stretch in the front-parallel plane; and the fit method, where the observer has to change the appearance of a ‘gauge figure’ so it fits the pictorial relief. The results show that:

- The metrical bi-dimensionality of shapes does not necessarily coincide with the perceived bi-dimensionality.
- Depth (remoteness) is a mental product that the observer can expand/contract idiosyncratically.
- Seeing and touching surfaces are not totally unrelated perceptions in extended subjective space [253].

Nevertheless, ways of defining the role and behavior of a surface in the flow continuum [6] and how they can be transformed into volumetric appearances still need to be understood, experimentally verified, and established.

### 9.3. Qualitative Notation

No matter how relevant all the initial studies on visual units may be, they are only drops in a vast ocean. Much work still needs to be conducted and in a clear-cut framework. The ways in which a point, line, surface (or volume) appear need to be considered, as well as their mutual transformation into dynamic visual configurations. There are several examples here: a visual point can transform into a line (such as a triangular or rectangular trajectory of a small fly); a curved line can become a linear one; a line can become a surface by closure; a concave surface can become a convex one in a certain light; a surface can become a volume (perceptual transparency); and what appears to be a closed volume can become a distant 2D surface [257], etc. All this depends on how they look and change when presented in certain durations, spatial locations, and perceived lights. Even the concept of categorial identity becomes questionable in these perceived changes. To paraphrase Monet: in a presentation, one aspect of visible nature contains it all.

To answer the question of how to construe a consequent geometry based on the appearances and behavior of phenomenological units and their relations would require a separate study. However, our aim in the future is to reach a definition of the visual points, lines, surfaces, and volumes that are susceptible to a qualitative notation (appearances that have a greater or lesser “pointness”, “surfaceness”, and so on). Verifying the neural correlates of these qualitative perceptions would be a complementary and interesting achievement, paving the way for a collaborative effort towards a geometry of the visible.

## 10. Phenomenal Continuum in Awareness

In the previous paragraphs, we discussed the specific qualitative nature of awareness, to be identified in the act part of the process of seeing; the (2) empirical and (3) internal factors on which it grounds; and what and where of objects as subjectively perceived in the visual field. This is only a minimal part of the complexity of consciousness.

A complete theory and model for the phenomenal continuum of the flow of consciousness is not yet available, and understandably so, due to the lack of an established psychological theory of consciousness, for example. However, it is precisely the non-resolution of this issue that leads us to accept the idea of consciousness (at primary and higher levels) as a discrete series of states [258] that can be represented on a continuum of dimensionless points or as states. This widespread concept continues to reduce the experience of the continuum to the discrete, while, as discussed, there are neither dimensionless instants nor dimensionless points in awareness. Also, continuity and discontinuity in physics and awareness do not coincide because, in seeing, physical discontinuity can become phenomenal continuity. Particularly striking demonstrations of this discrepancy are shown by the Tunnel effect [40,106,210,214], the Renard effect [259], the Window effect [156,260], the Stopping effect [156], and the Transition effect [261].

A very interesting phenomenon in this respect also concerns the perception of a continuous change in brightness, where mixed perceptions between continuity and discontinuity may occur between the continuity of a change and the evident difference between two different states of brightness in the same object [262]. These phenomena, which have a rather short range of duration (between approximately 100 and 200 ms), offer a series of information, including:

- The demonstration of the different kinds of continuity/discontinuity in (Newtonian) physics and awareness.
- The different qualitative types of phenomenal movements (partial, pure, hindered, etc.).
- The invalidity in the phenomenal field of the strict before-after relationship generally holds for physical facts.
- A series of identifiable specific durations and
- The optimal intervals are required to perceive a fluid continuous movement.

Having said this, the current challenge is how to devise and experimentally develop a visual continuum of consciousness theory. Phenomenological philosophy offers some lines of thought [263], while several aspects of the phenomenal continuum have already been highlighted by the above-mentioned experimental outcomes. In any case, before addressing the issue of the phenomenal continuum, there are certain preliminary conditions to consider, such as:

- Avoid starting from abstract definitions.
- Put the immediate stimuli quantification in brackets and enter (and remain) within the domain of qualities and their internal relationships.
- Consider how to ground the structure of the phenomenal continuum in an interwoven series of durations and spatial localizations, where one sequential chain of physical factors can give rise to two or more simultaneous chains of perceptual events.
- Be aware of the intrinsic aesthetic quality of the domain, especially in terms of the balance, conflict, and equilibrium of the phenomenal field’s boundary forces, which are also responsible for the expressive value of their dimensions [264,265,266].
- Recognize that the phenomenal continuum has different modes, which are strictly dependent on the subjective space–time relationships shaping the phenomena.

For example, in the Tunnel effect, one sees the continuous movement of an object, but in certain (larger) intervals, one sees two separate objects, each of which moves individually [129,210]. The latter is a case of consecutive continuum, which does not display the same kind of intermediacy. In experimental terms, the boundary of the phenomena regarding single-moving objects is consecutively continuous. The opposite is the case for the Stereokinetic effect (see above, Section 5), where in the continuum of their deployment, the non-independent parts (ellipse, ring, disc) touch each other and become one. This is a case of continuous movement, in which the boundaries of two objects touch and become fused into a single boundary that belongs to both parts. The phenomenon of fusion (Verschmelzung), as analyzed in music perception [158,267,268,269], also applies to visual configurations of color and light. In these phenomena, boundaries become smooth transitions. More simply, take an orange and blue line; the boundary between the two-colored parts can be sharp or smooth and may behave differently with darker or lighter colors. Many examples of different kinds of perceptual boundaries are represented beautifully in Rothko’s paintings (for boundaries in pictorial space, see [270]). Whatever the kind of boundaries that divide or connect visual phenomena, the appearances are always qualitatively evaluated by the viewer in terms of “more or less”, for example, “more or less continuous”, “more or less conspicuous”, “more or less sharp”, “more or less smooth”, “more or less fast”, “more or less two-dimensional” and so on. As we have seen, several known phenomena can highlight a boundary’s different ways of being in the phenomenal continuum, but a specific experimental focus on it and its organization in a system is still lacking in the phenomenological field itself, and further studies and tests are required. Finding out the neural correlates of the phenomenal continuum might shed light on both the differences and the correlations between the two ways of processing visual information.

## 11. Conclusions

For centuries, to build a science and a scientific explanation of perception, we have moved forward on land considered safe: physics. In so doing, we carefully avoided sinking into the sands of subjective experience, which was not explainable by the protocols of the “scientific” requirements. Still, to ultimately, reduce the complexity of our subjective experiences (awareness, first) to physics is a no-go.

The challenge that the many different approaches to vision (see Section 1) have encountered has been how to avoid matching perception on both the physical stimulus and the cognitive judgments. Gestalt psychology and experimental phenomenology gave abundant evidence that escaping from this quicksand is possible and that an explanation of subjective experience at the phenomenological level is scientifically feasible, verifiable, and measurable [71].

In my opinion, the root of the partial failure of the different approaches in perception science, in many respects, has been a willful ignorance of the role and value of subjectivity (i.e., of first-person perception) and its relationship with the qualitative environment or biological niche we perceive. This negligence has also surfaced in the study of vision, which is the most developed research in this field. Psychophysics and, more recently, neuroscience have obtained or are continuing to obtain increasingly relevant information on physical and neurophysiological processes. However, while the main aim of psychophysics is to describe how a physical continuum of a certain type maps at the perceptual level, in my opinion, neuroscience’s “sin of pride” resides in its insistence on seeking the “ultimate” explanation of psychic phenomena in the functioning of the brain. The scientific achievements of the latter have led to the conception of models that explain all the aspects of human perception and cognition using the same theories, tools, and methods, often featured in a model of mankind copied from machine design. My contribution started from the consideration that, notwithstanding the differences, psychophysical, neurophysiological, and phenomenological approaches in vision science are focused on visual percepts, leaving aside the issue of self-awareness, which is the dimension allowing a subject to perceive. To address this issue, I argued, one should identify the specific factors of awareness, which I singled out in subjective space–time factors. To support the thesis, results from the experimental phenomenological research have been provided, discussed, and related to neurophysiological findings.

Then, how to proceed? The collaboration between experimental phenomenologists and neuroscientists is highly recommended once the latter agrees that neurophysiological brain analysis cannot univocally explain qualitative experiences in a first-person account. Libet’s work is a valuable example showing that the relationship between the brain and consciousness is addressable and fruitful. There is a lot that could result from a fruitful collaboration in both research fields on topics that still need to be addressed and developed in vision science. I mentioned, for example, the identification of the dimensions of the perceptual continuum and the development of a geometry of the visible starting from its visual “elements”. Many other aspects of visual perception are on the waiting list.

## Data Availability

The original contributions presented in the study are included in the article and Supplemental Materials, further inquiries can be directed to the corresponding author.

## Supplementary Materials

The following supporting information can be downloaded at: https://www.mdpi.com/article/10.3390/brainsci14070735/s1, Figure S1. Michotte’s parallelepiped. Figure S2. Bistable rectangle. Figure S3. The rotating ellipse.

## Appendix A

**Table A1:** Benussi, 1913 [154].

Durations Very short (90 ms to 234–252 ms) Short (234–252 ms to 585–630 ms)
**Indeterminate** (the most vivid) (585–630 ms) to (1080–1170 ms) (time of contemporaneity) Long (1080–1170 ms to 2070 ms) Very long → 2070 ms.

**Table A2:** D’Angiulli & Reeves, 2021 [221].

Durations 50 ms (successive events cannot be separated, i.e., there is simultaneity) very short (90 to 107 ms) short (234 to 229 ms) (successive events can be separated with difficulty). Subjective attitude: *Analysis* intermediate (585 to 490 ms) (successive events can be separated with difficulty)
**long (1080 to 1048, 6). Subjective attitude of *Focus* (major vividness)** (time of contemporaneity) very long (2070 to 2244 ms) (successive events can be integrated with difficulty). Subjective attitude: *Synthesis* extended (4500 to 4802, 2 ms) (successive events are always perceived as separate) 4500 (specious present or STM memory) (James) <12 s.

**Table A3:** Window effect and Stopping effect.

**Window effect** (from Vicario, 2005, 59–62 [129]). - if st > 275 ms you see the rectangular surface enter the window, pause for a moment, and resume movement until it disappears. - if 275 > st > 130 ms you see a moving rectangular surface enter the window, stop and then start again at the same moment it stopped. - if 130 st > 0 you see a rectangular surface pass through the window with a uniform and constant movement.
**Stopping effect (a variant of the Renard effect)** (from Vicario, 2005, 57–59 [129]) A small white rectangle starts from a position on the right, moves to pass under a screen, and then manages to return to its initial position. To see the moving rectangle stop, the stop must physically last for at least 200 msec. Stops between 200 and 65 ms offer a vision of the movement in two stages. Stops between 65 and 30 ms offer a vision of uninterrupted but non-linear movement. Stops between 30 and 0 ms are not seen.
