# Supplementary material for: An Unavoidable Mind-Set Reversal: Consciousness in Vision Science
Source: Brain Sci. 2024 Jul 22;14(7):735. doi: 10.3390/brainsci14070735 (PMC11274483; doi:10.3390/brainsci14070735)
Supplement: Supplementary file 1 [file brainsci-14-00735-s001.zip › brainsci-3067835-supplementary.pptx]

## Slide 1
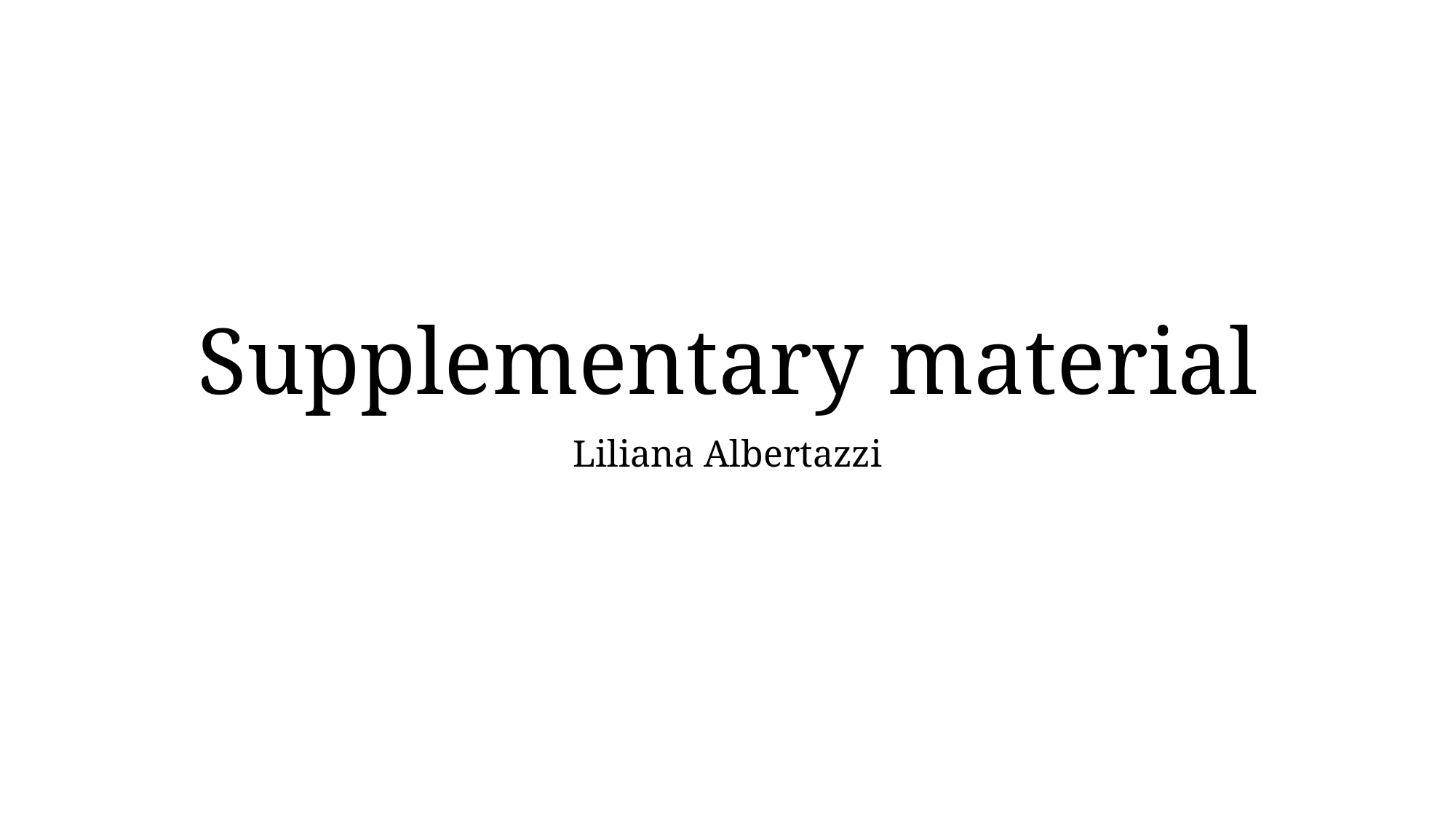

# Supplementary material
Liliana Albertazzi

## Slide 2
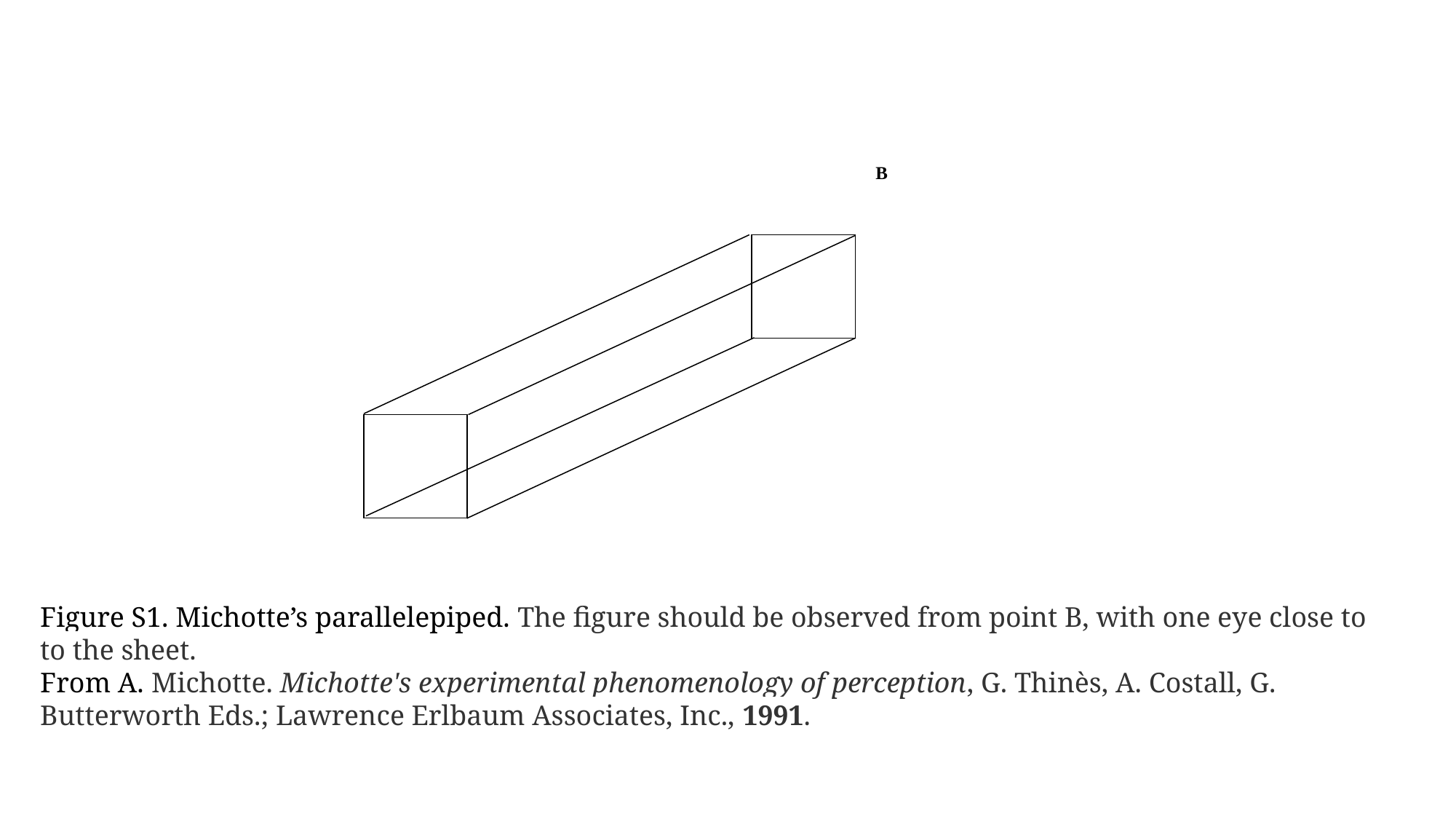

B
Figure S1. Michotte’s parallelepiped. The figure should be observed from point B, with one eye close to to the sheet.
From A. Michotte. Michotte's experimental phenomenology of perception, G. Thinès, A. Costall, G. Butterworth Eds.; Lawrence Erlbaum Associates, Inc., 1991.

## Slide 3
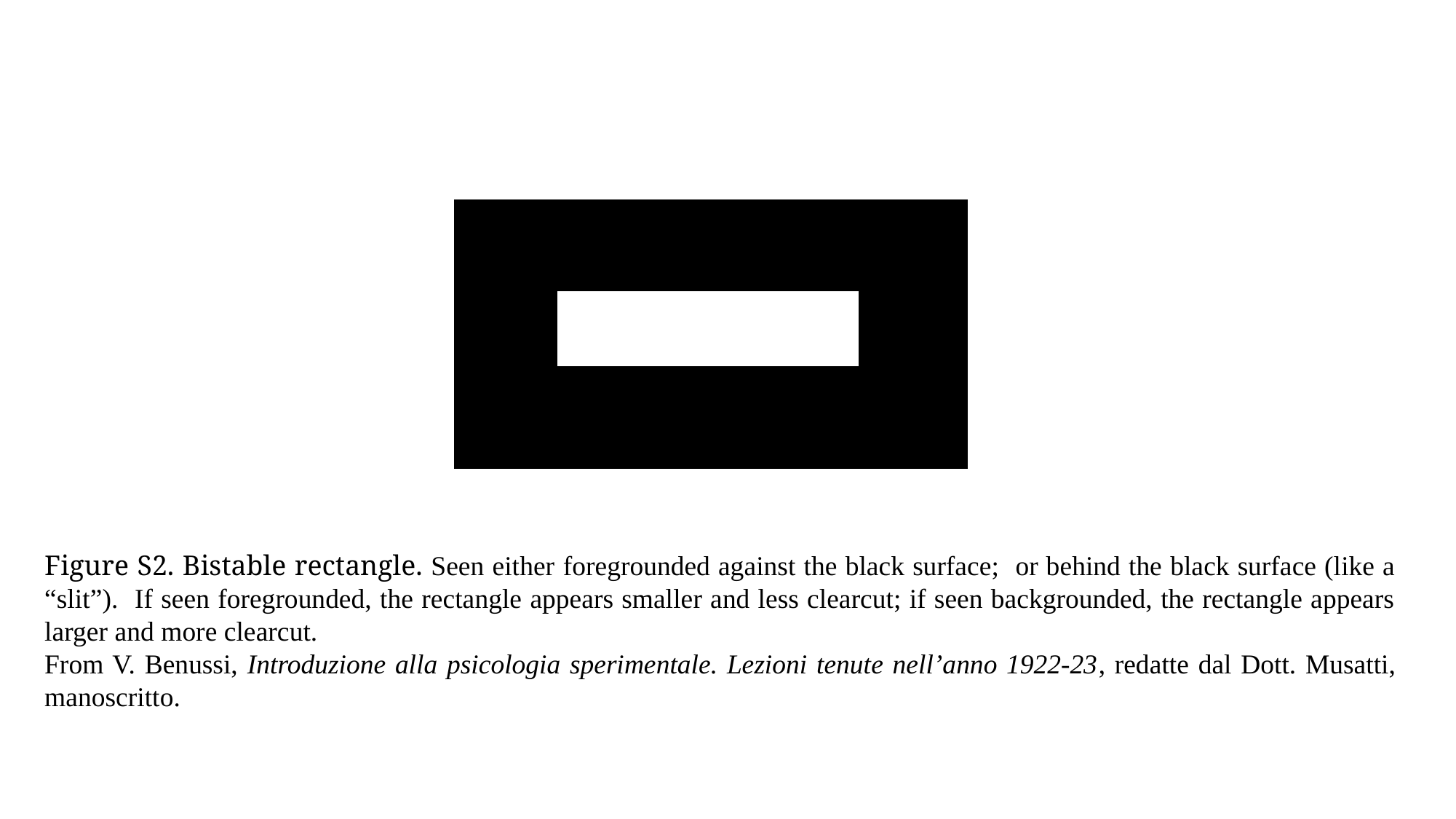

Figure S2. Bistable rectangle. Seen either foregrounded against the black surface; or behind the black surface (like a “slit”). If seen foregrounded, the rectangle appears smaller and less clearcut; if seen backgrounded, the rectangle appears larger and more clearcut.
From V. Benussi, Introduzione alla psicologia sperimentale. Lezioni tenute nell’anno 1922-23, redatte dal Dott. Musatti, manoscritto.

## Slide 4
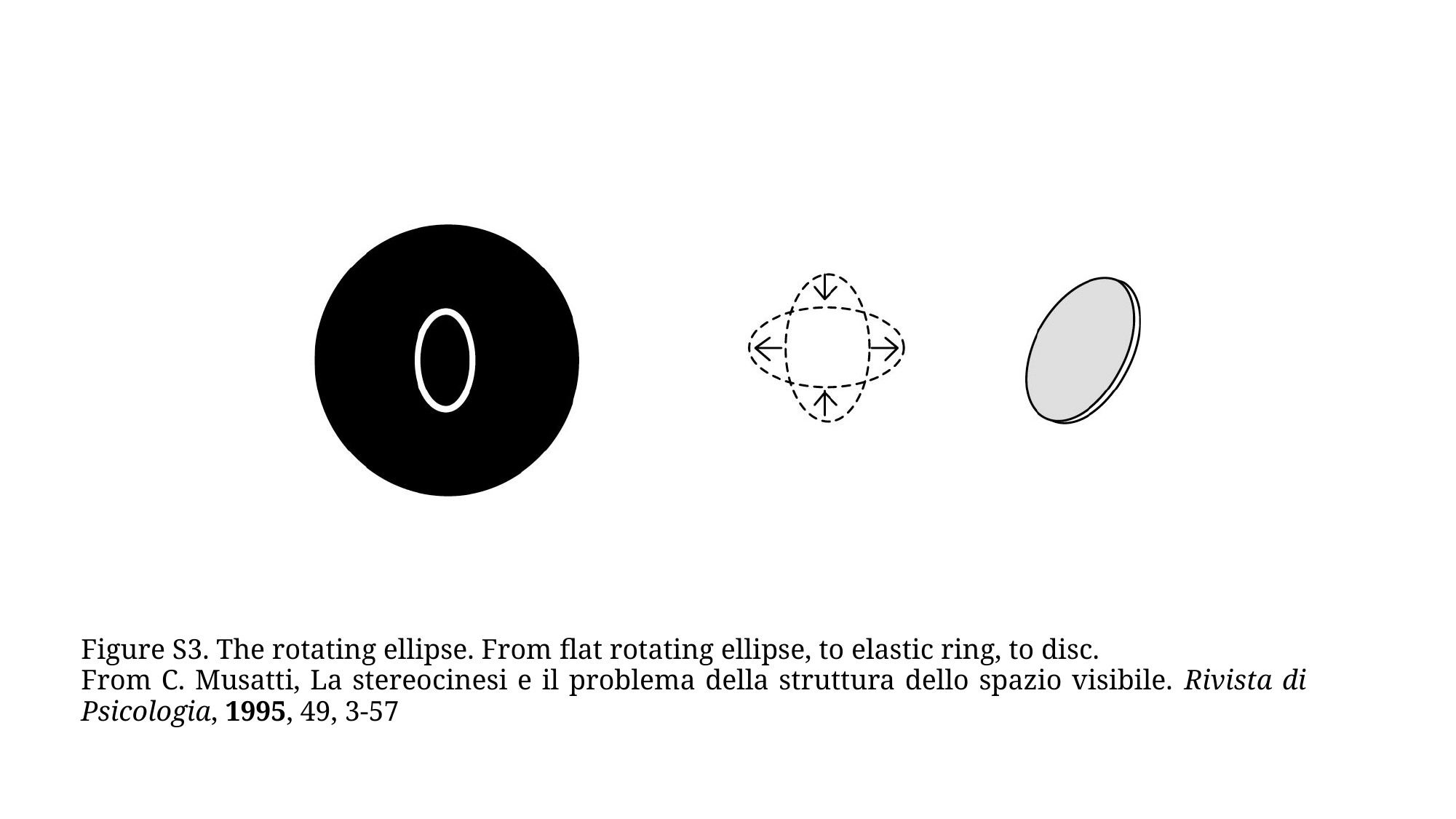

Figure S3. The rotating ellipse. From flat rotating ellipse, to elastic ring, to disc.
From C. Musatti, La stereocinesi e il problema della struttura dello spazio visibile. Rivista di Psicologia, 1995, 49, 3-57
